# Special Analytical and Health Commission on Tinned and Preserved Food

**Published:** 1906-09-15

**Authors:** 


### SPECIAL ANALYTICAL AND HEALTH COMMISSION ON TINNED AND PRESERVED FOOD.

### SWEETNESS AND SUGAR.

In our last article we discussed, *inter alia*, the question of saltness, the subject of the present monograph appeals to another equally fundamental taste—namely, sweetness. To the average person the idea of sweetness is inseparably associated with sugar, and indeed with the sugar cane or cane sugar. It is true other substances possess sweetness, some to a much greater degree than cane sugar. Among these may be mentioned saccharin and dulcin. It is computed that saccharin is 500 times as sweet as sugar, and that one part of it will impart sweetness to 70,000 times its weight of water. If we compare from the double standpoint, however, these substances with sugar we find they are frauds—veritable wolves masquerading in sheeps' clothing. They possess the shadow without the substance; they are sweet, but, unlike sugar, are incapable of yielding energy to the body, have no nutritive value, and are in no sense foods. Saccharin and dulcin were christened to emphasise the fact that they possess one property possessed by sugar, but chemically they are as far removed from sugar as they are dietetically; saccharin, for instance, is an aromatic compound, being an imide of toluene sulphuric acid.

### Varieties of Sugar.

All flesh is not the same flesh, and all sugar is not the same sugar, but all sugar, and, so far as we know, all starch, is converted by the body into one variety of sugar before it is absorbed—namely, dextrose. Of all sugars, cane sugar is the sweetest. Chemically it is a disaccharose, and, according to the Pharmacopœia, should be obtained from the juice of the sugar cane; but it can also be obtained from the beet and certain other plants. The juice constitutes about 90 per cent. of the cane, and 11 per cent. of it is extracted and actually becomes crystallisable sugar. After a somewhat elaborate technical process a clear syrup is obtained from the juice; this is concentrated until crystallisation takes place, and the crystals are subsequently separated by centrifugalisation from the mother liquor. The crystals become refined sugar, and the mother liquor becomes treacle and molasses. A point of interest and importance with regard to the properties of cane sugar is that if acid solutions of it are boiled it becomes changed by hydrolysis, and equal parts of two other sugars are formed—namely, laevulose and dextrose. This substance containing equal parts of the two last-named sugars is technically known as invert sugar. This substance is much less sweet than cane sugar, and for this reason cooks and jam-makers always avoid the prolonged boiling of fruit syrups or such like preparations. A sugar which occurs naturally in the animal body, and which, although not so sweet as cane sugar, belongs chemically to the same class, is lactose, or sugar of milk, of which ordinary milk contains about 6 per cent. An important characteristic of sugar is the ease with which it is split up by ferments into alcohol, water, and carbon dioxide. The disaccharoses, cane sugar, and milk sugar are not directly fermentable by yeast, but become so after hydrolysis. In this respect cane sugar is inferior to lactose or milk sugar, for, as we have seen, cane sugar is converted into laevulose and dextrose, both fermentable by ordinary yeast, whereas lactose is split up into dextrose and galactose, the latter—not fermentable to ordinary yeast; hence it not infrequently happens that cane sugar will be followed by gastric fermentation, and will disagree, while milk sugar will not.

### Nutritive Value of Sugar.

Sugar, with perhaps the single exception of alcohol, is the most labile form of energy that an ordinary diet contains. Directly the body has modified the kind of sugar ingested, or in other words, has transformed it into dextrose, this substance is at once available as a source of energy to the body. As proteids or meat foods are especially associated with the keeping of the framework of the body in repair, as fats seem to bear an intimate relation to the keeping up of the body temperature, the sugars are pre-eminently concerned in supplying the energy for muscular work. Those readers of Xenophon's "Anabasis" who have physiological minds will no doubt remember with interest that the great general's emergency ration consisted largely of honey, which is essentially invert sugar. Modern experiments upon the marching of troops have confirmed the value of sugar as a food. The heart can be kept alive and doing work outside the body if it is simply transfused with a mineral solution containing sugar and oxygen.

### Cheapness of Sugar.

Sugar is not only of extreme nutritive value, but it is absolutely the cheapest source of energy we possess. If it were possible for a working man to nourish himself upon sugar he could live for 3d. per day; for three pennyworth of sugar would supply approximately the number of calories required by him per diem, or the same amount as would be supplied by half a bottle of brandy.

### Jam.

Dietetically, if we exclude starch, our most common and extensive source of sugar is jam. Jam contains approximately 20 per cent. of cane sugar. Jam consists essentially of fruit rendered sterile by boiling and kept sterile by syrup or a strong solution of cane sugar. Economically, one of the most important functions of sugar is its action as a preservative, and here we also have an instance of the extraordinary effect of dose upon the characteristics of a substance; while almost nothing ferments more easily than a dilute solution of sugar, almost nothing resists fermentation so well as a strong syrup. As the action of syrup in preventing decomposition is unusual, and is similar to that exerted by glycerine and alcohol, it is worth considering. A syrup preserves by dehydration. In jam-making the fruit is initially rendered sterile by the temperature to which it is exposed. Any mould or fermenting agent which may subsequently gain access to the jam is at once killed by having the moisture in its cells, as it were, sucked out by the syrup, which possesses a very strong affinity for water. The action of salt and glycerine as preservatives is exactly the same as that of sugar, and is a bio-physical, and not a bio-chemical, process. Ideal jam should contain nothing but fresh fruit, water, and cane sugar, which latter, as we have seen above, is partially converted into invert. The public, however, dislike their jam when they keep it to deposit crystals of sugar, and to become so-called candied. Strong syrups made of cane sugar are very apt to do this, and to avoid this, and also for reasons of economy, commercial glucose is used as a partial substitute for cane sugar. Such jam is perfectly nutritious and perfectly wholesome. The public also like so-called whole fruit jams. Now if in jam-making boiling or temperature is the only means adopted to render the product sterile two phenomena are apt to occur. One is that the fruit—especially delicate fruit like raspberries and strawberries—are apt to get pulped and broken up, and the jam becomes very concentrated, or, in other words, it loses a very large quantity of water, which for trade reasons is hardly fair on the manufacturer, having in view the exceedingly low price at which jam has to be put upon the market. To avoid the necessity for this very prolonged boiling, and to fortify the preservative properties of the article, some jam-makers use a very small, and in the opinion of the author a harmless, quantity of some chemical preservative—generally salicylic acid. The public also like their jam of a bright, attractive colour. Practically all natural colours are unfavourably influenced by heat, and hence it is often deemed advisable to add to jam some artificial colouring matter. One does not like to eat jam that has not a good fresh colour any more than one likes white milk, white butter, or white cheese, and the artificial colouring of the one is quite as harmless as that of the other.

### Samples Received.

Thanks to the kindness of the leading jam-makers, we have received several samples of fruit jams. Messrs. James Keiller and Sons, of Dundee, have forwarded us samples of their whole-fruit black currant jam, their strawberry jam, and their Dundee marmalade. This firm is well known throughout the world for the excellency and purity of their marmalade and jams. The samples sent us are without doubt of first-class quality, and we have no hesitation whatever in being able to accord to them unqualified praise. Messrs. William P. Hartley, Liverpool and London, have sent us two specimens of their marmalade. The first one we may describe as a pure marmalade of exceedingly good quality, made from fresh gathered Seville oranges and the finest sugar only. The second variety is what is termed jelly marmalade, and practically consists of a jelly having the taste of fresh oranges, containing practically no solid constituents. We can well imagine, and have, indeed, reason to know, that such a preparation as this latter is exceedingly useful to those who, while requiring the natural laxative properties of marmalade, are unable to stand the solid portions of it. The same firm have also submitted to us specimens of their raspberry and red currant, black currant and strawberry jams. We find all these of good quality, carefully prepared, and distinctly to be recommended. Messrs. Richard Dickeson and Co., the well-known Army contractors, have kindly forwarded a glass jar of their strawberry and of their black currant jams. These jams are made from fresh fruit, keep well after the jar is opened, and possess a good fresh flavour. To Messrs. Chivers and Sons, Histon, Cambridge, we are indebted for specimens of their raspberry jam and of their strawberry jam. As is well known, the jam of this firm is made from fruit grown in their own fruit plantations. The samples before us are both of unquestionably first quality, and both in appearance and taste leave little to be desired. The Croom Estate Jam Co., of Pershore, have sent us a sample of the fresh plums from which their jam is made. The fruit, as we received it, was dry, sound, of good quality and taste. With regard to the finished product we have to thank them for a glass jar of raspberry, strawberry, and apricot jam. All these are well made and thoroughly reliable jams. The Kent Jam Co., of Swanley, Kent, have forwarded samples of their black currant and of their raspberry jams. Situated as this company is, practically in the midst of the great fruit-growing district of the country, their opportunity of obtaining good, fresh, and dryly gathered fruit must be unrivalled. We have every reason to believe, in fact, the samples submitted to us show that they utilise to the fullest their advantage and produce in every way a wholesome and nutritious jam.

